# Supplementary material for: Thymidine Analogue Mutations with M184V Significantly Decrease Phenotypic Susceptibility of HIV-1 Subtype C Reverse Transcriptase to Islatravir
Source: Viruses. 2024 Dec 6;16(12):1888. doi: 10.3390/v16121888 (PMC11680407; doi:10.3390/v16121888)
Supplement: Supplementary file 1 [file viruses-16-01888-s001.zip › viruses-3293672-supplementary.pdf]

Table S1. Reference sequences of laboratory-adapted strains.

Subtype	Strain	GenBank Accession Number
B	DS9	MH234640 [65]
	LTNP5	DQ009850 [66]
	SM1	AY835755 [67]
	SM2	KF469956 [68]
C	CM9	AF411967 [69]
	DU151	AF544009 [70]
	DU179	AF544000 [71]
	DU422	AF544005 [70]

Table S2. NRTI exposure of PLWH from which sequences were obtained.

NRTI exposure	3TC	ABC	AZT	d4T	FTC	TDF
**Current regimen**	41.9% (5,486)	11.3% (1,474)	26.1% (3,415)	0.8% (101)	6.9% (901)	13.0% (1,702)
**Previous regimen**	33.7% (3,494)	8.3% (860)	8.3% (860)	16.0% (1,658)	10.8% (1,123)	23.0% (2,386)

3TC: lamivudine; ABC: abacavir; AZT: zidovudine; d4T: stavudine; FTC: emtricitabine; TDF: tenofovir disoproxil fumarate. Please note that total numbers are greater than n = 7,749 since patients could have received two or more NRTIs in a single regimen.

Table S3. Sequences of primers used for site-directed mutagenesis to synthesize single and combination mutation profiles.

Introducing mutation	Base plasmid	Primer	Primer sequence (5’ → 3’)
A62V	p8.9MJ4	Forward	AATAAAAAAGAAAGACAGTACTAAGTG
	M184V	Reverse	ACAAATACTGGAGTATTATATGGATTTTCAG
A62V	K65R/M184V	Forward	AATAAAAAGGAAAGACAGTACTAAG
		Reverse	ACAAATACTGGAGTATTATATGGATTTTCAG
D67N	M184V	Forward	CAGTACTAAGTGGAGAAAATTAGTAGAC
		Reverse	TTTTTCTTTTTTATTGCAAATACTGGAG
D67N	K70E/M184V	Forward	CAGTACTGAGTGGAGAAAATTAGTAGAC
		Reverse	TTTTTCTTTTTTATTGCAAATACTGGAG
K70T	p8.9MJ4	Forward	ATGGAGAAAATTAGTAGACTTCAGGG
		Reverse	GTAGTACTGTCTTTCTTTTTTATTGCAAATAC
K70T	K65R/M184V	Forward	GTGGAGAAAATTAGTAGACTTCAGGG
		Reverse	GTAGTACTGTCTTTCCTTTTTATTGCAAATAC
L74I	p8.9MJ4	Forward	AGTAGACTTCAGGGAACTTAATAAAAGAAC
	M184V	Reverse	ATTTTTCTCCACTTAGTACTGTCTTTC
L74V	p8.9MJ4	Forward	AGTAGACTTCAGGGAACTTAATAAAAGAAC
	M184V		
	p8.9NSX*	Reverse	ACTTTTCTCCACTTAGTACTGTCTTTC
Y115F	p8.9MJ4	Forward	TTTTTCAGTTCCTTTAGATGAAGGC
	K65R/M184V		
	L74V/M184V	Reverse	AATGCATCCCCCACATCTAATACTGTTAC

A114S	p8.9MJ4 M184V	Forward	ATATTTTTCAGTTCCTTTAGATGAAG
		Reverse	GAATCCCCCACATCTAATACTGTTACTG
T215Y	M41L/M184V	Forward	CACACCAGACAAGAAACATCAGAAAGAAC
		Reverse	TAAAATCCCCACTTTAATAGATGGTTTC
K219E	K65R/M184V	Forward	GAAACATCAGAAAGAACCCCCATTTC
		Reverse	TCGTCTGGTGTGGTAAATCCCCACTTTAATAG
K219Q	D67N, K70R, M184V	Forward	GAAACATCAGAAAGAACCCCCATTTC
		Reverse	TGGTCTGGTGTGGTAAATCCCCACTTTAATAG

The p8.9MJ4 subtype C backbone was used to introduce a single mutation into.
*The p8.9NSX subtype B backbone was used as the base plasmid to introduce L74V.

Table S4. The prevalence of the top 20 combination NRTI mutations in patients with NRTI drug-resistant mutations.

Combination mutation profiles	Frequency	
	n	%*
L74V, Y115F, M184V	164	2.97
K65R, M184V	130	2.35
L74V, M184V	89	1.61
D67N, K70R, M184V, K219Q	87	1.58
A62V, K65R, M184V	79	1.43
D67N, K70R, M184V, K219E	77	1.39
M41L, M184V, T215Y	72	1.30
K70E, M184V	61	1.10
M41L, M184V	60	1.09
D67N, M184V	58	1.05
A62V, M184V	56	1.01
K65R, M184V, K219E	46	0.83
D67N, K70E, M184V	44	0.80
M184V, T215Y	43	0.78
K65R, Y115F, M184V	40	0.72
L74I, M184V	38	0.69
M41L, M184V, T215F	37	0.67
K65R, K70T, M184V	37	0.67
D67N, K70R, M184V	32	0.58
D67N, K70R, M184V, T215F, K219E	28	0.51
A114S, M184V**	-	-

* (% $= \frac{x}{5\,521} \times \mathbf{100}$)
** This mutation profile (and the single mutation A114S) was not in this database; however, a recent study showed that the A114S/M184V mutation combination reduced susceptibility to ISL [16]. Therefore, it was included in this study.

Table S5. The prevalence of the individual mutations that constitute the top 20 combination NRTI mutations in patients with NRTI drug-resistant mutations.

Mutation list	Frequency			
	Single		In combination	
	n	%*	n	%*
M184V	2 105	38.13	3 004	54.41
D67N	26	0.47	992	17.97
K65R	18	0.33	724	13.11
K219Q	11	0.20	391	7.08
A62V	6	0.11	315	5.71
K219E	6	0.11	484	8.77
M41L	4	0.07	630	11.41
K70R	4	0.07	758	13.73
L74V	4	0.07	413	7.48
T215Y	4	0.07	370	6.70
K70E	2	0.04	303	5.49
L74I	2	0.04	287	5.20
K70T	0	0.00	101	1.83
A114S**	0	0.00	0	0.00
Y115F	0	0.00	518	9.38
T215F	0	0.00	340	6.16

*(n = $\frac{x}{5\,521} \times \mathbf{100}$)

**A114S is an in vitro ISL-selected drug-resistant mutation.

Table S6. Median IC_{50}- and FC values of single NRTI-resistant mutants in subtype C.

Mutation	Median IC_{50} (IQR) (nM)	Fold change			Fold change in literature**
		Median (IQR)	Dunn's multiple comparisons test p-value*	Significant p-value	
M41L	10.19 (7.60 – 27.90)	1.23 (0.91 – 3.35)	>0.999	No	0.8 – 2.1 [16]
A62V	23.22 (15.01 – 40.83)	2.79 (1.81 – 4.91)	0.182	No	-
K65R	2.94 (0.73 – 5.10)	0.35 (0.09 – 0.61)	>0.999	No	0.2 [8,10] – 0.4 [16,44]
D67N	12.43 (2.79 – 14.29)	1.49 (0.34 – 1.72)	>0.999	No	-
K70E	1.66 (1.65 – 3.81)	0.20 (0.20 – 0.46)	0.956	No	-
K70R	10.45 (6.21 – 15.02)	1.26 (0.75 – 1.81)	>0.999	No	-
K70T	11.65 (11.15 – 15.54)	1.40 (1.34 – 1.87)	>0.999	No	-
L74I	6.97 (6.11 – 7.85)	0.84 (0.73 – 0.94)	>0.999	No	0.9 – 1.4 [16]
L74V	21.72 (6.92 – 34.34)	2.61 (0.83 – 4.13)	0.895	No	0.2 [8,10]
A114S	18.01 (14.04 – 21.14)	2.17 (1.69 – 2.54)	0.530	No	1.9 – 2.2 [16,45]
Y115F	17.38 (13.47 – 23.36)	2.09 (1.62 – 2.81)	0.299	No	-
M184V	38.53 (27.23 – 45.01)	4.63 (3.27 – 5.41)	0.006	Yes	5.0 [8,16,40] – 78.9 [8,10,16,39,40]
T215F	3.73 (3.38 – 3.99)	0.45 (0.41 – 0.48)	>0.999	No	-
T215Y	25.94 (21.70 – 30.39)	3.12 (2.49 – 3.65)	0.025	Yes	2.5 [16]
K219E	7.36 (5.01 – 19.45)	0.89 (0.60 – 2.34)	>0.999	No	-
K219Q	2.48 (1.72 – 9.88)	0.30 (0.21 – 1.19)	>0.999	No	-

* Significance was calculated by comparing the median fold change to the fold change of the p8.9 WT using Dunn's multiple comparisons test.

** The Stanford HIV Drug Resistance Database (https://hivdb.stanford.edu/) provides predictions for drug resistance; however, this is not yet available for ISL. Hence, as the final mode of analyzing the single mutation PSVs' FC values, publications were consulted to see if our phenotypic data corresponded with published data.

Table S7. Intra-subtype comparison of fold-change values of the L74V mutant in laboratory-adapted HIV-1 PSVs in response to ISL.

Subtype	Strain	Median IC_{50} (IQR) (nM)	Fold change		
			Median (IQR)	Dunn's multiple comparisons *p-value	Significant p-value ($p < 0.05$)
B	**p8.9NSX-WT**	7.92 (2.55 – 14.06)	0.93 (0.30 – 1.65)	-	-
	**p8.9NSX-L74V**	6.30 (4.13 – 7.65)	0.74 (0.48 – 0.90)	>0.999	No
	**DS9-L74V**	2.31 (1.78 – 4.20)	0.27 (0.21 – 0.49)	0.171	No
	**LTNP5-L74V**	9.76 (6.27 – 9.98)	1.14 (0.73 – 1.17)	>0.999	No
	**SM1-L74V**	5.99 (5.43 – 8.10)	0.70 (0.64 – 0.95)	>0.999	No
	**SM2-L74V**	7.03 (5.52 – 7.16)	0.82 (0.65 – 0.84)	>0.999	No
C	**p8.9MJ4-WT**	7.27 (4.16 – 12.88)	0.87 (0.50 – 1.55)	-	-
	**p8.9MJ4-L74V**	21.72 (6.92 – 34.34)	2.61 (0.83 – 4.13)	0.445	No
	**CM9-L74V**	5.42 (4.49 – 6.19)	0.65 (0.54 – 0.64)	>0.999	No
	**DU151-L74V**	4.68 (4.45 – 5.35)	0.56 (0.54 – 0.64)	>0.999	No
	**DU179-L74V**	4.88 (2.44 – 6.63)	0.59 (0.29 – 0.80)	>0.999	No
	**DU422-L74V**	3.82 (0.53 – 4.60)	0.46 (0.06 – 0.55)	0.196	No

* Significance was calculated by comparing the median fold change to the fold change of the respective subtype's p8.9 WT using Dunn's multiple comparisons test.

Table S8. Median IC_{50}- and FC values of combination NRTI-resistant mutants in subtype C.

Mutation profile	Median IC_{50} (IQR) (nM)	Fold change			Fold change in literature
		Median (IQR)	Dunn's multiple comparisons test p-value*	Significant p-value	
**Non-TAM**					
A62V, M184V	52.19 (44.54 – 64.83)	6.28 (5.36 – 7.79)	0.028	Yes	-
K65R, M184V	18.92 (17.81 – 25.75)	2.28 (2.14 – 3.10)	>0,999	No	2.1 [8]
A62V, K65R, M184V	11.84 (9.72 – 13.49)	1.42 (1.17 – 1.62)	>0,999	No	-
K65R, K70T, M184V	38.58 (25.51 – 41.50)	4.64 (3.07 – 4.99)	0.486	No	-
K65R, Y115F, M184V	18.66 (13.31 – 32.31)	2.24 (1.60 – 3.88)	>0,999	No	-
K65R, K70E, M184V	20.76 (17.48 – 25.95)	2.50 (2.10 – 3.12)	>0,999	No	2.3 [8]
L74I, M184V	78.69 (44.13 – 103.50)	9.46 (5.13 – 12.45)	0.016	Yes	-
L74V, M184V	22.69 (10.63 – 32.46)	2.73 (1.28 – 3.90)	>0,999	No	2.3 [8]
L74V, Y115F, M184V	35.55 (31.74 – 44.09)	4.28 (3.82 – 5.30)	0.348	No	-
A114S, M184V	>tested	>60[ǂ]	0.0002	Yes	25 – 38 [16,45]
**TAM-1**					
M41L, M184V	34.96 (29.41 – 43.00)	4.20 (3.54 – 5.17)	0.486	No	5.6 [16]
M184V, T215Y	49.75 (31.69 – 84.58)	5.98 (3.81 – 10.17)	0.066	No	-
M41L, M184V, T215F	77.00 (61.74 – 116.30)	9.26 (7.42 – 13.98)	0.001	Yes	-
M41L, M184V, T215Y	36.29 (20.91 – 44.21)	4.36 (2.51 – 5.32)	0.219	No	-
**TAM-2**					
K65R, M184V, K219E	40.06 (30.89 – 44.38)	4.82 (3.17 – 5.34)	0.315	No	-
D67N, M184V	79.82 (44.99 – 107.70)	9.60 (5.41 – 12.95)	0.010	Yes	-
D67N, K70E, M184V	44.93 (31.70 – 79.91)	5.40 (3.81 – 9.61)	0.088	No	-
D67N, K70R, M184V	48.11 (48.04 – 51.68)	5.78 (5.78 – 6.21)	0.034	Yes	-
D67N, K70R, M184V, K219E	129.10 (103.10 – 147.80)	15.52 (12.39 – 17.77)	0.001	Yes	-
D67N, K70R, M184V, K219Q	149.70 (139.30 – 194.40)	18.00 (16.75 – 23.38)	0.001	Yes	-
D67N, K70R, M184V, T215F, K219E	157.50 (103.50 – 210.90)	18.94 (12.45 – 25.36)	0.001	Yes	-

The median IC_{50}- and FC values were calculated using the average WT MJ4 IC_{50} value of 8.3 nM (± 5.0 nM).

[ǂ]60 was the maximum FC value possible, as the highest concentration of ISL tested (0.5 µM) divided by the average WT MJ4 IC_{50} value of 8.3 nM = 60.

* Significance was calculated by comparing the median fold change to the fold change of the p8.9 WT using Dunn's multiple comparisons test
